# What is needed to achieve HCV microelimination among HIV-infected populations in Andalusia, Spain: a modeling analysis

**DOI:** 10.1186/s12879-020-05285-z

**Published:** 2020-08-08

**Authors:** Britt Skaathun, Annick Borquez, Antonio Rivero-Juarez, Sanjay R. Mehta, Francisco Tellez, Manuel Castaño-Carracedo, Dolores Merino, Rosario Palacios, Juan Macías, Antonio Rivero, Natasha K. Martin

**Affiliations:** 1 Division of Infectious Diseases and Global Public Health, University of California San Diego, 9500 Gilman Drive MC 0507, La Jolla, CA 92093 USA; 2 Infectious Diseases Unit, Instituto Maimonides de Investigaciones Biomedicas de Cordoba (IMIBIC), Hospital Universitario Reina Sofia de Cordoba, Universidad de Cordoba, Cordoba, Spain; 3 Infectious Diseases Unit Hospital Universitario de Puerto Real, Instituto de Investigación e Innovación en Ciencias Biomédicas de la Provincia de Cádiz. Universidad de Cádiz, Cádiz, Spain; 4 Infectious Diseases Unit, Hospital Regional Universitario de Málaga, Málaga, Spain; 5 Infectious Diseases Unit. Hospitales Juan Ramón Jiménez e Infanta Elena de Huelva, Huelva, Spain; 6 Infectious Diseases Unit, Hospital Universitario Virgen de la Victoria. Complejo Hospitalario Provincial de Málaga, Málaga, Spain; 7 Unidad de Enfermedades Infecciosas, Hospital Universitario de Valme. Instituto de Biomedicina de Sevilla (iBiS), Sevilla, Spain; 8 Population Health Sciences, University of Bristol, Bristol, UK

**Keywords:** Hepatitis C virus, Prevention, Microelimination, HIV, Direct-acting antivirals

## Abstract

**Background:**

Scale-up of hepatitis C virus (HCV) treatment for HIV/HCV coinfected individuals is occurring in Spain, the vast majority (> 85%) with a reported history of injecting drug use and a smaller population of co-infected men who have sex with men (MSM). We assess impact of recent treatment scale-up to people living with HIV (PLWH) and implications for achieving the WHO HCV incidence elimination target (80% reduction 2015–2030) among PLWH and overall in Andalusia, Spain, using dynamic modeling.

**Methods:**

A dynamic transmission model of HCV/HIV coinfection was developed. The model was stratified by people who inject drugs (PWID) and MSM. The PWID component included dynamic HCV transmission from the HCV-monoinfected population. The model was calibrated to Andalusia based on published data and the HERACLES cohort (prospective cohort of HIV/HCV coinfected individuals representing > 99% coinfected individuals in care in Andalusia). From HERACLES, we incorporated HCV treatment among diagnosed PLWH of 10.5%/year from 2004 to 2014, and DAAs at 33%/year from 2015 with 94.8% SVR. We project the impact of current and scaled-up HCV treatment for PLWH on HCV prevalence and incidence among PLWH and overall.

**Results:**

Current treatment rates among PLWH (scaled-up since 2015) could substantially reduce the number of diagnosed coinfected individuals (mean 76% relative reduction from 2015 to 2030), but have little impact on new diagnosed coinfections (12% relative reduction). However, DAA scale-up to PWLH in 2015 would have minimal future impact on new diagnosed coinfections (mean 9% relative decrease from 2015 to 2030). Similarly, new cases of HCV would only reduce by a mean relative 29% among all PWID and MSM due to ongoing infection/reinfection. Diagnosing/treating all PLWH annually from 2020 would increase the number of new HCV infections among PWLH by 28% and reduce the number of new HCV infections by 39% among the broader population by 2030.

**Conclusion:**

Targeted scale-up of HCV treatment to PLWH can dramatically reduce prevalence among this group but will likely have little impact on the annual number of newly diagnosed HIV/HCV coinfections. HCV microelimination efforts among PWLH in Andalusia and settings where a large proportion of PLWH have a history of injecting drug use will require scaled-up HCV diagnosis and treatment among PLWH and the broader population at risk.

## Background

In 2016, the World Health Organization (WHO) released elimination targets including an 80% reduction in new hepatitis C virus (HCV) infections and 65% reduction in HCV related mortality by 2030 [1]. Indeed, there is considerable optimism HCV elimination is possible due to the advent of highly-effective, short duration, tolerable, all-oral HCV direct-acting antiviral therapies (DAAs) which can cure > 90% of HCV monoinfected and HIV/HCV coinfected individuals alike, and which modeling studies indicate could prevent onwards transmission at a population level [2–12]. In response, numerous countries are developing or have developed plans to scale-up HCV treatment to achieve these elimination targets, and focused efforts at microelimination (elimination among targeted populations, settings, or limited geographical areas) among key populations such as people living with HIV (PLWH) have been developed. For example, clinical societies such as the British HIV Association have set HCV microelimination targets among HIV+ populations [13, 14], yet how to achieve microelimination is unclear. Indeed, recent studies highlighting observations of dramatic reductions in HCV incidence observed among HIV+ men who have sex with men (MSM) in the Netherlands alongside widespread DAA scale up [15], combined with modeling studies indicating HCV elimination may be achievable among PLWH in France [16], have fuelled optimism that microelimination among PLWH is achievable.

In 2015, Spain developed a National Strategy for HCV treatment and instituted widespread scale-up of HCV DAA therapies for individuals coinfected with HIV and HCV, the vast majority (> 85%) with a reported history of injecting drug use, and the remainder MSM or unknown [17]. PWID are the main group at risk for HCV infection in Spain, and the main group at risk for prevalent and incident HIV/HCV coinfection [17–19]. In 2015, a multicentre prospective observational cohort of HIV-infected patients with chronic HCV infection from 21 reference centers in Andalusia, Spain was established (HERACLES cohort). From 2015 to 2017, the HERACLES cohort was used to assess HCV treatment uptake and outcomes among HIV/HCV coinfected patients, since implementation of the Spanish National Strategy [17, 20]. As such, it provided important data with which to examine the potential impact of this treatment scale-up on HCV incidence among PLWH and the broader population.

Numerous theoretical epidemic modeling studies have explored the potential of HCV treatment for prevention in a range of global settings [5–8]. Despite several studies modeling HIV and HCV coinfection transmission among PWID [21–23], and HCV prevention among HIV-positive MSM [24–27], to our knowledge no published study has explored how to achieve microelimination within HIV+ individuals when the vast majority of new infections are among PWID. One study examined elimination among HIV+ individuals in France [16], but did not incorporate full transmission dynamics from HIV-negative PWID, which could contribute substantially to infection/reinfection among HIV-infected individuals with ongoing injecting risk. Additionally, that study did not examine the impact of treatment scale-up to coinfected individuals on the broader HCV epidemic among PWID.

We use dynamic epidemic modeling to assess the population impact of HCV treatment scale-up to HIV-infected individuals on HCV incidence among PLWH and the broader population, and implications for achieving the WHO HCV elimination target of 80% reduction in incidence by 2030, using Andalusia, Spain as a case study.

## Methods

### Mathematical model

**Fig. 1:**
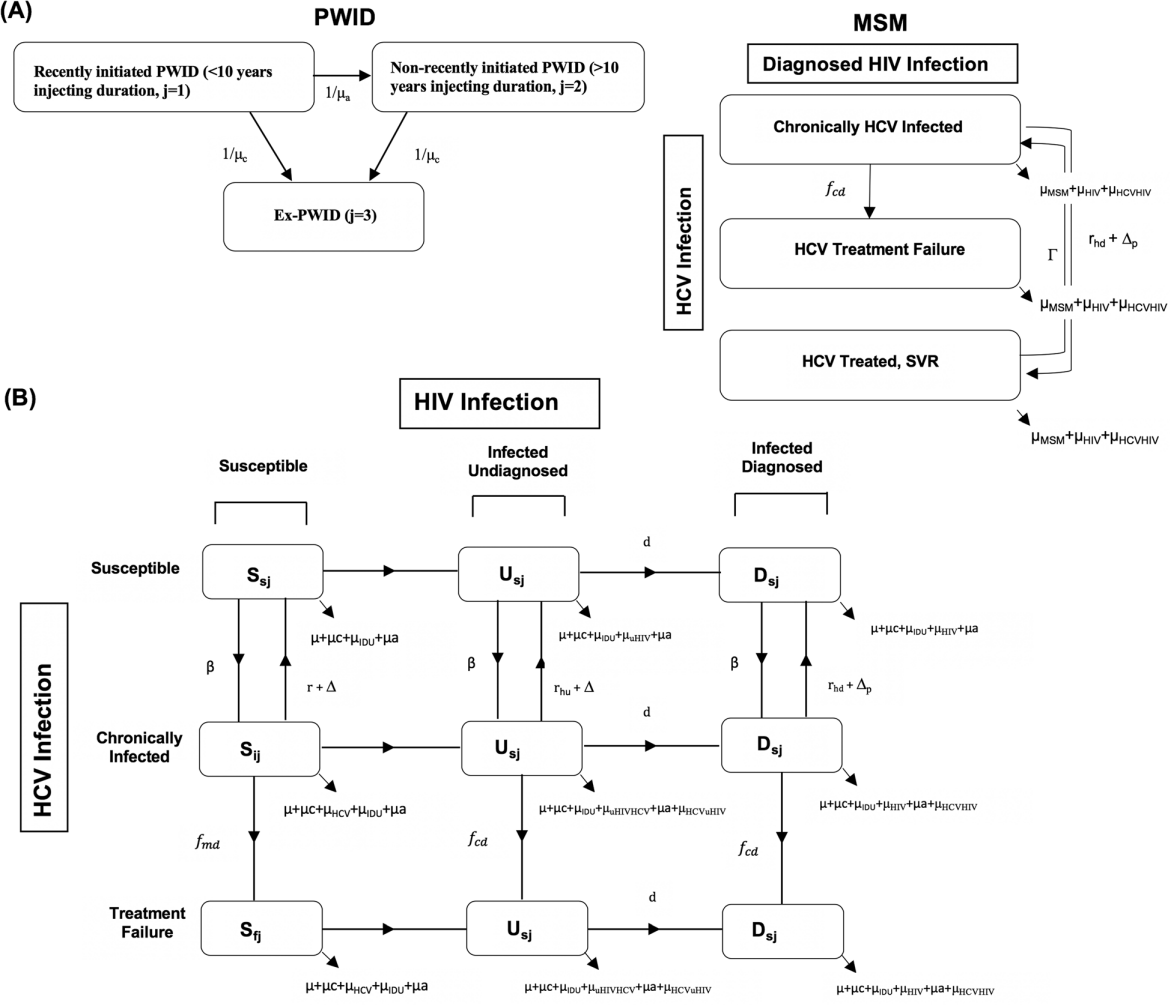
Model schematics showing (**a**) stratification by population and (**b**) HIV and HCV transmission and treatment stages for the PWID component. PWID: people who inject drugs. MSM: men who have sex with men. HCV: hepatitis C virus. SVR: sustained viral response

A deterministic dynamic coinfection model was developed, and calibrated to Andalusia, Spain. The model was stratified by PWID and MSM risk groups (Fig. 1a). The PWID component included dynamic HCV transmission from the HCV-monoinfected population, and therefore a joint HIV and HCV transmission model was developed to simulate the transmission of HIV and HCV due to injecting (HIV and HCV) and sexual risk (only HIV) among PWID (Fig. 1b). The joint model incorporated infection with HIV only (uninfected, HIV-infected undiagnosed, HIV-infected diagnosed and in care), HCV only (uninfected, HCV chronically infected (RNA+), HCV chronically infected treatment failure), and HIV/HCV coinfection (stratified by the above characteristics). Due to observed differences in HIV and HCV prevalence by injecting status and duration, the model was additionally stratified by history of injection drug use with PWID who initiated injecting < 10 years ago (j = 1), PWID who initiated injecting > 10 years ago (j = 2) and ex-PWID (j = 3). The model is open, such that PWID continually enter the “PWID who initiated <10yrs ago” compartment, and leave all stages through cessation of injecting or death. We assume HCV transmission only occurs among PWID; ex-PWID do not contribute to transmission. HIV injecting-related transmission occurs among PWID, and HIV sexual transmission occurs between all PWID and ex-PWID. As 97% of HIV-infected individuals who are diagnosed and in care are on HIV antiretroviral therapy (ART) in Spain [28], and 97% of HIV/HCV coinfected individuals in Andalusia are on ART [17] we make the simplifying assumption that all individuals who are HIV diagnosed and in care are on ART. We assumed that HIV-infected PWID can become diagnosed and in care, where they are at decreased probability of injecting and sexual HIV transmission compared to the undiagnosed stage due to receipt of ART. As per clinical practice in Spain, we assume that all individuals who are diagnosed with HIV will be tested for HCV. Finally, the model incorporates biological interactions between HCV and HIV infection, such that HIV-infection reduces the probability of HCV spontaneous clearance (Table 1) [2, 32]. Additionally, we assumed HIV infection increases HCV transmissibility through elevated HCV viral loads if the individual is not on ART [46, 47, 58, 59]. As the vast majority of newly diagnosed and prevalent HIV/HCV coinfections are among PWID, we utilize a simplified model of HCV/HIV coinfection among MSM, stratifying the model by HCV treatment status (treatment naïve, failed treatment, achieved SVR and are at risk of reinfection) and incorporated a fixed incidence of infection and reinfection. As there are currently no restrictions on retreatment after reinfection in Spain, we allow retreatment for individuals who are reinfected after successful treatment. Individuals who fail treatment (do not attain sustained viral response SVR) are not retreated.

**Table 1:** Model parameters and Sources

**Calibration Data**				
**Calibrated parameter**	**Value and Source**			
Number of diagnosed and in care HIV-infected and chronic HCV coinfected (HIV+/HCV+) in 2015	3075 as observed in the HERACLES cohort [17], with 2669 [range 2402–2936] with a history of PWID			
HCV chronic prevalence among PWID injecting for < 10 years in 2010	45% [sampled uniformly from 40 to 50%]. Values from Folch et al. [29], adjusted for weighted spontaneous clearance given HIV coinfection prevalence and spontaneous clearance rates by HIV-status in the below table.			
HCV chronic prevalence among PWID injecting for > 10 years in 2010	60% [sampled uniformly from 55 to 65%] Values taken from Folch et al. [29], adjusted for weighted spontaneous clearance given HIV coinfection prevalence and spontaneous clearance rates by HIV-status in the below table.			
HIV prevalence among PWID injecting for <10 years, in 2010	20% [sampled uniformly from 10 to 30%] Values from Folch et al. [29]			
HIV prevalence among PWID injecting for > 10 years in 2010	40% [sampled uniformly 30–50%]. Values from Folch et al. [29]			
HCV chronic prevalence among HIV+ ever PWID in 2015	54% [sampled uniformly 50–58%] [18]			
Proportion of HIV+/HCV+ diagnosed individuals who are current PWID in 2015	14% [sampled uniformly 10–18%] as observed in the HERACLES cohort [17]			
Proportion HIV+/HCV+ diagnosed ever PWID who had previously failed HCV treatment in 2015	32% [sampled uniformly 30–34%] as observed in the HERACLES cohort [17]			
**General Parameters**				
**Parameter Definition**	**Symbol**	**Sampled Value and 95% Interval**	**Sampling Distribution**	**Source**
Sustained HCV viral response, monoinfected on DAAs	1- *f*_*md*_	0.9		[2]
Sustained HCV viral response, coinfected on DAAs	1- *f*_*cd*_	0.948		HERACULES cohort
Sustained HCV viral response, monoinfected pre-DAAs	1- *f*_*mpd*_	0.52 (0.45–0.60)	Uniform [0.45–0.60]	Pooled SVR weighted by genotype and genotype distribution [30, 31].
Sustained HCV viral response, coinfected pre-DAAs	1- *f*_*cpd*_	0.30 (0.20.-0.39)	Uniform [0.20–0.40]	Pooled SVR weighted by genotype and genotype distribution [30, 32].
Excess death rate due to mono-infection with HIV untreated per year	μ_uHIV_	0.097 (0.09–0.10)	Uniform [1/9.6–1/11.5]	[33]
Decreased mortality hazard ratio for HIV on ART	μ_HIV_	0.26 (0.20–0.32)	Uniform [1/5–1/3]	[34, 35]
Excess death rate due to chronic HCV mono-infection per year	μ_HCV_	0.00169 (0.0016–0.00181)	Uniform [0.00154–0.00183]	[36]
Relative risk of death due to chronic HCV for HIV/HCV coinfection not on HIV treatment compared to HCV monoinfection	RR__co_noART_	2.58 (1.70–3.99)	Lognormal, from distribution 2.5 (95%CI 1.8–3.4)	[36, 37]
Excess death rate due to HCV for HIV-HCV co-infection with no HIV treatment per year	μ_HCVuHIV_	0.0042 (0.0039–0.0046)	Uniform [0.0038–0.0046]	[36, 37]
Relative risk of death due to chronic HCV for HIV/HCV coinfection on HIV treatment compared to HIV/HCV coinfection no HIV treatment	RR__coART_		0.68	[36, 37]
Excess death rate due to HIV for HIV-HCV co-infection with no HIV treatment per year	μ_uHIVHCV_	0.0005 (0.00041–0.00066)	Uniform [0.0004–0.00069]	[36]
PWID parameters				
Inflow of new PWID per year	*θ*			Deaths replaced, rate is halved in 2011. See text for discussion.
Population size of active PWID in 2010		7,500 (5,000-10,000)	Uniform [5,000-10,000]	8.4 million Andalusia population in 2010, 92% > age 15, and a PWID prevalence among adults of 0.1% (national estimate) [38, 39],
HCV treatment rate among PWID who are HIV- or HIV+ and undiagnosed (%/year)	*r*, *r*_*hu*_	0.99% (0.54–1.44%)	Uniform [0.5–1.5%]	Assumed similar to estimates worldwide [40]
HCV treatment rate among HIV+ diagnosed individuals prior to 2015 (%/year)	*r*_*hd*_	Calibrated		Calibrated to fit HERACLES data on proportion previously failed treatment in 2015
HCV treatment rate among HIV+ diagnosed individuals from 2015 to 2020 (%/year)	*r*_*hd*_	33%		HERACLES cohort (*unpublished data from 2015 to 2017)*
Average duration of injecting until final cessation (years)	1/μ_cd_	15.1 (6.8–24.4)	Uniform [5–25]	Uncertain, so vary widely [41, 42]
Background mortality rate per year among PWID	μ	0.0179		Average life expectancy in Spain to 80.7 (WHO life table), and average age of injecting initiation age of 25 [43]
Overdose mortality rate among PWID per year	μ_IDU_	0.0062 (0.005–0.007)	Uniform [0.0053–0.0070]	[44]
Relative risk of HIV transmission through sex for those on ART	RR_HIVs_	0.107 (0.03–0.198)	Lognormal [95% CI 0.01–0.27]	[45]
Relative risk of HIV transmission through injecting for those on ART	RR_HIVi_	0.50 (0.27–0.72)	Uniform [0.25–0.75]	Limited data. One modeling study in Vancouver estimated 44% efficacy for preventing injecting transmission [5] but uncertainty was wide.
Relative risk of HCV transmission if HIV positive undiagnosed (off ART) compared to HIV negative	RR_HCV_ifHIVpos_	2 (1.2–2.8)	Uniform [1–3]	[46, 47]
Annual probability of HIV transmission through sex during the latent untreated stage of infection	β_HIVs_ = 1/6* β_HCV_			[21]
Proportion spontaneous HCV clearance among HIV negatives	∆_n_	0.25 (0.22–0.29)	Uniform [0.22–0.29]	[48]
Relative risk of spontaneous clearance among HIV positives compared to HIV negatives	RR^sponclearHIVpos^	0.68 (0.39–0.90)	Lognormal [0.46–1.0]	[49]
MSM parameters				
Number of HIV/HCV coinfected and diagnosed MSM in 2015		406		HERACLES cohort (*unpublished)*
Number of newly HIV/HCV coinfected and diagnosed MSM each year		Calculated		Based on multiplying the number of HIV-infected MSM in 2010 by the proportion of HIV+ MSM who are diagnosed, by the primary HCV incidence among HIV-infected MSM.
Number of HIV-infected MSM in 2010	*θ*_*MSM*_	16,281 (9,018–23,526)	Uniform [8,646-23,913]	[50–52] Based on multiplying the male population over 15 years of age by the prevalence of MSM, and the prevalence of HIV-infected MSM in 2010
Proportion of HIV+ MSM who are diagnosed		0.761 (0.751–0.771)	Uniform [0.75–0.772]	[53]
Primary HCV incidence among HIV-infected MSM (per 100 person-years)		1.17 (0.75–1.16)	Uniform [0.73–1.61]	[54] See supplement for details.
HCV reinfection incidence among HIV+ MSM (per 100 person-years)	Γ	7.07 (3.87–10.27)	Uniform [3.7–10.44]	[55]
Background (non HIV or HCV) mortality rate among HIV/HCV-diagnosed MSM (per year)	μ_MSM_	0.026		Average life expectancy in Spain of 80.7 [56], and average age at HIV/HCV diagnosis of 43 [57].

### Model setting and data

The model was parameterized by and calibrated to data from Andalusia, Spain. Information on the diagnosed HIV/HCV coinfected population in Andalusia was obtained from the HERACLES cohort (clinicaltrial.gov: NCT02511496), a prospective cohort of 3075 HIV-infected individuals with chronic HCV infection from 21 reference hospitals set up in March 2015, and followed for 24 months [17]. Of them, 2669 patients (86.7%) had a reported previous or current history of injecting drug use. These individuals were drawn from 19 centers treating 15,556 HIV-infected patients, which represent 99.3% of the HIV-infected patients in the Andalusia Health care system. Information such as age, route of HIV and HCV transmission, genotype, history of HCV therapy, and opiate substitution therapy (OST) use was collected. Individuals in the cohort were followed-up every 3 months, with the main objective of evaluating the current status and follow-up of chronic HCV infection in patients coinfected with HIV in Andalusia. Information from the cohort was used to obtain the following data for the model: the number of HIV individuals diagnosed with chronic HCV and with a history of injecting drugs in 2015, the proportion of HIV/HCV diagnosed individuals who are PWID, the proportion of HIV-infected ever PWID with chronic HCV infection who previously failed treatment in 2015, the HCV treatment uptake rate among HIV/HCV coinfected individuals with a history of injecting from 2015 to 2017.

The model was calibrated to the following data from the HERACLES cohort: 3075 diagnosed HIV+/HCV+ coinfected individuals in 2015. Of these, 2669 had a history of injecting drug use, among whom an estimated 14% were current PWID (defined as receipt of OST or current injecting risk. Thirty-two percent were treatment experienced and who had failed previously therapy. Additionally, the model was calibrated to the following epidemiological data from Spain: chronic HCV prevalence of 45 and 60% among PWID who initiated injecting < 10 years ago and > 10 years ago, respectively in 2010 [29], HIV prevalence of 20 and 40% among PWID who initiated injecting < 10 years ago and > 10 years ago, respectively in 2010 [29], chronic HCV prevalence among HIV+ ever PWID (PWID and ex-PWID) of 54% in 2015 [18], and HCV reinfection incidence among HIV+ MSM of 5.93/100 person-years [55]. Due to a lack of available data, we used global pooled estimates of HCV primary incidence among HIV+ MSM (see Table 1 and supplement for details) [18, 54, 55].

Several data sources coincide in indicating a reduction in the numbers of people newly initiating injecting drug use over the past decade in Andalusia and across Spain more broadly. First, data from OST admissions in Andalusia indicates a sharp drop among people with a history of heroin use in 2011 (from approximately 4000–4600 per year across 2005–2010 and then declining to 2400–2800 per year from 2011 to 2015 (via the Indicador Admisiones a Tratamiento 2015). At the same time, national surveillance data indicates a drop in the contribution of PWID to new HIV infections between 2004 and 05 and 2010–11, which could be attributable to population changes such as a reduction in people newly initiating injecting drug use leading to fewer newly initiated PWID at risk instead of lower transmission risk [28, 60]. Given these data, we incorporated a reduction in the entry rate of new PWID starting in 2011 (by 50% based on a halving of entrants to OST in Andalusia starting in 2011 onwards).

Given uncertainty in the data underlying the model’s parameterization and calibration, we sample parameters and generate multiple model fits. We sampled most parameters from underlying distributions (Table 1) using Latin Hybercube Sampling a total of 500 times. The model was calibrated to the sampled epidemiological data detailed above (HCV and HIV prevalence among PWID by duration of injecting, HCV prevalence among HIV+ ever-PWID, number diagnosed HIV/HCV coinfected MSM and ever-PWID, proportion diagnosed HIV/HCV coinfected ever-PWID who are current PWID, and proportion of diagnosed HIV/HCV coinfected ever-PWID who had previously failed HCV treatment). The model was calibrated using an optimization solver with multiple start points minimizing the least squares (MATLAB lsqnonlin solver using the MultiStart function), and selecting fits which lay within the uncertainty range for HCV and HIV prevalence among PWID from the data. This resulted in 134 selected model fits which were used for the full model simulations.

### HCV treatment

#### Pre DAA treatment rates (prior to 2015)

We assumed HCV treatment started in 2004, at a low rate of 1% among HCV moninfected PWID consistent with other global settings [61]. We calibrated the HCV treatment rate among diagnosed HIV/HCV coinfected individuals to fit the HERACLES data on proportion who had previously failed treatment in 2015 (no difference was observed between risk groups).

### Observed DAA scale-up (from 2015)

Consistent with observations in the HERACLES cohort and more broadly among HIV-infected cohorts in Spain, we model a scale-up of HCV DAA therapy to HIV-diagnosed individuals from 2015 onwards. In the first 24 months of DAA availability from 2015, 63% of HIV/HCV coinfected individuals within the HERACLES cohort in Andalusia were treated (*unpublished data from 2015 to 2017*). The proportion of individuals treated was significantly lower among PWID at 55% (defined as on OST or with a history of injecting < 10 years) compared to ex-PWID or MSM 70%. Given these data, we incorporate a HCV treatment rate of 33%/year among diagnosed HIV/HCV coinfected PWID, ex-PWID and MSM from 2015 onwards. Observed SVR rates of 94.8% among the HERACULES cohort were used. We assume treatment rates among HCV monoinfected PWID remained stable at pre-2015 values.

#### HCV Treatment Scale-Up Scenarios from 2020

We model the impact on HCV incidence and chronic prevalence among PLWH and overall until 2030 with the following scenarios:

1. **Status quo:** Continuing current treatment rates (33%/year among diagnosed HIV/HCV coinfected individuals from 2015 as observed in the HERACLES cohort from 2015 to 2017)
2. **Coinfected scale-up from 2020:** 100% screening and treatment of coinfected individuals
3. **All PWID scale-up from 2020:** As in [2] plus HCV treatment of HCV moninfected PWID of 10%/yr
4. **No scale-up from 2015** (counterfactual scenario of continuation of historic treatment rates of 10.5% among diagnosed HIV/HCV coinfected individuals)

#### Univariate sensitivity analyses

Multiple univariate sensitivity analyses were performed to test the sensitivity of results to parameter assumptions on the relative reduction in the annual number of newly diagnosed HIV/HCV coinfections in scenario 3 (all PWID scale-up from 2020). Due to uncertainty in future SVR rates, we evaluate the impact of lower DAA SVR (85 and 90% vs. 95% at baseline). Additionally, because there is uncertainty in the PWID entry rate, and whether the observed declines in first time entrants to OST reflect true population reductions in new initiates to injecting, we also evaluate a scenario where the rate of injecting initiation did not decline in 2010.

## Results

### Epidemic prior to DAA scale-up in 2015

The model fit well to the HIV and HCV calibration data (Supplementary Figure 1 ), producing model runs, which represent the range of uncertainty in our data. The annual number of newly diagnosed HIV/HCV coinfections (defined as new HCV infections among HIV-diagnosed as well as new diagnoses of HIV among HCV-infected individuals, some who may have acquired their HCV prior to their HIV infection) was projected to be 200 infections (2.5–97.5% interval 139–299) in 2015, the majority (84%) among those with a history of injecting drug use. Among PWID, we project that HCV chronic prevalence was increasing among PWID in Andalusia due to a reduction in the number of PWID initiating injecting, leading to an aging cohort with higher burden of disease (Supplementary Figure 3). In 2015, an estimated mean of 46% of all prevalent HCV infections and an estimated mean of 12% of incident HCV infections among PWID were among HIV+ PWID.

### Modeled impact of observed DAA scale-up since 2015

**Fig. 2:**
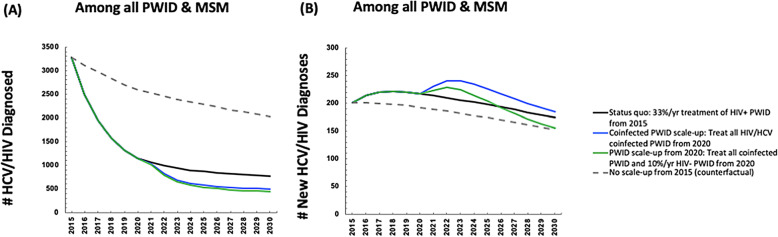
Mean model projections of the (**a**) number of HIV/HCV diagnosed individuals (**b**) number of new HIV/HCV diagnosed individuals with observed scaled-up DAA treatment rates from 2015 in Andalusia with various treatment scale-up scenarios

Observed scale-up of DAAs to HIV-diagnosed individuals since 2015 (from an estimated 10.5%/year to 33%/year as observed in the HERACLES cohort) likely reduced the number of diagnosed coinfected individuals by 12% (mean 3289 in 2015 to 1324 in 2019 Fig. 2a). However, the number of newly diagnosed HIV/HCV coinfections was projected to increase, from a mean of 200 in 2015 to 221 in 2019 (Fig. 2b) due to reinfection after successful treatment among those with ongoing risk.

Continuing current DAA treatment rates among HIV-diagnosed individuals could dramatically reduce the number of prevalent diagnosed coinfected individuals (mean 76% relative reduction (95%I: 75–76) from 2015 to 2030, Fig. 2a, black line), moderately reduce the number of newly diagnosed HIV/HCV coinfections (mean 12% relative reduction (95%I: 10–15) from 2015 to 2030, Fig. 2b, black line), and would moderately increase the number of new HCV infections (diagnosed and undiagnosed) among PLWH (mean − 12% relative reduction (95%I: − 38 - 36) 2015–2030). When considering the impact on the broader HCV epidemic, the model projects continuing current rates would reduce the number of new HCV infections by a relative 29% (95%I: 19–37%) by 2030.

**Fig. 3:**
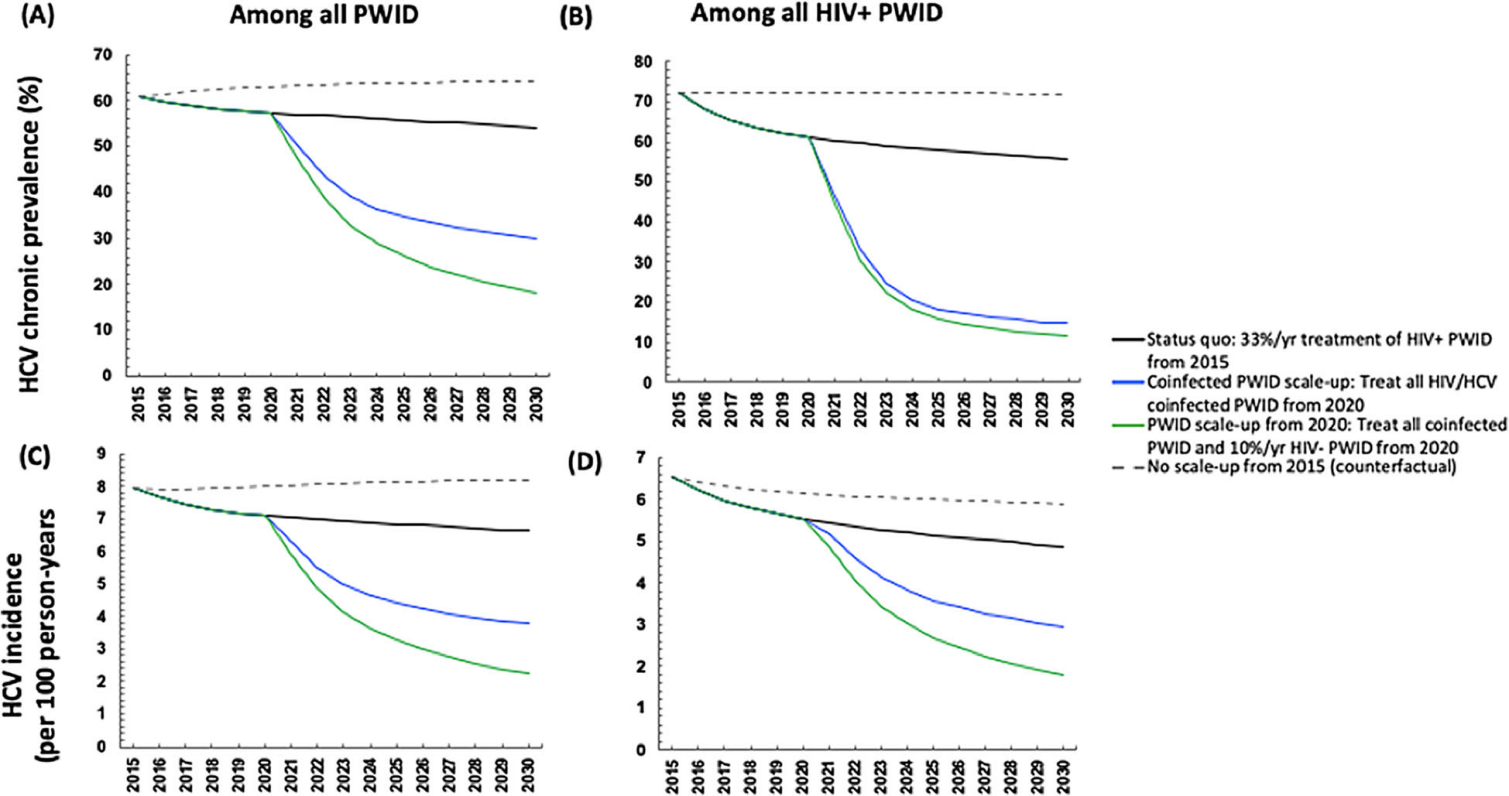
Mean model projections of the (**a**) number of chronic HCV infections (**b**) number of new chronic HCV infections with observed scaled-up DAA treatment rates from 2015 in Andalusia with various treatment scale-up scenarios

Among PWID, (Fig. 3), the scale-up was projected to marginally reduce both chronic HCV prevalence (mean relative reduction of 5% [95%I: 1–7%]) and incidence rates among PWID (mean relative reduction of 9% [95%I: − 0.09 to 10%]) from 2015 to 2019. Negative lower bounds correspond to scenarios where HCV chronic prevalence slightly increased from 2015 to 2019 due to background population dynamics. Without scale-up of treatment from 2015, the model projects that HCV incidence and prevalence in 2019 would have been higher (Fig. 3, dashed grey line).

### Modeled future impact of further scale-up of DAA treatment among HIV/HCV coinfected individuals

If all coinfected individuals were diagnosed and treated annually from 2020, this could lead to an 85% (95%I: 83–86%) relative reduction from 2015 in number of diagnosed coinfected individuals by 2030. However, this would only reduce the annual number of new diagnosed coinfections by 8% (95%I: 7–16%) by 2030. (Fig. 2b, blue line).

**Fig. 4:**
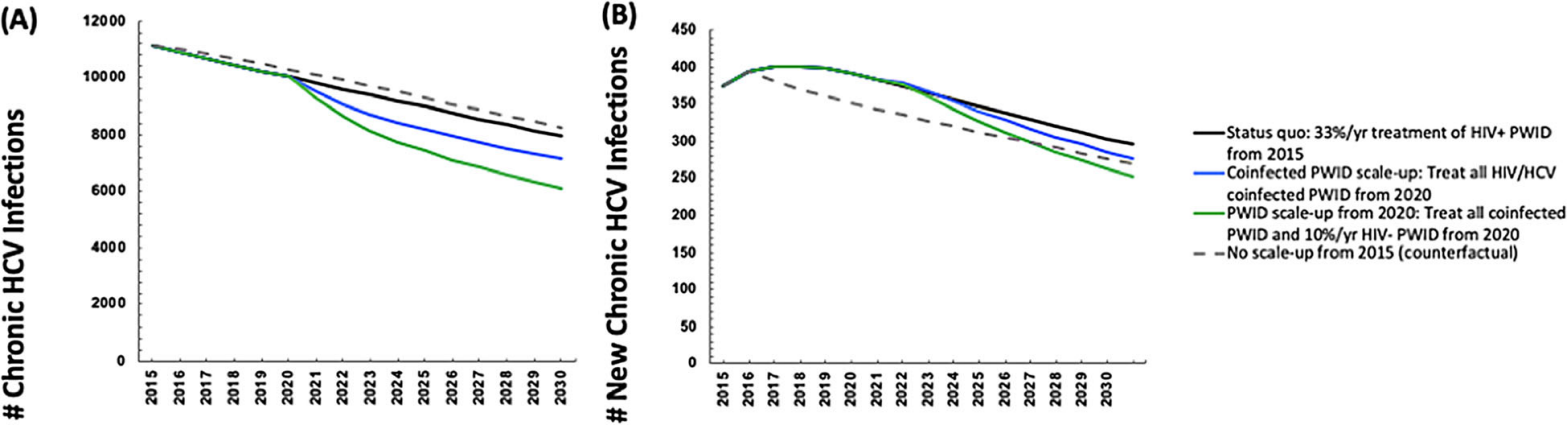
Mean model projections for HCV chronic prevalence and incidence among people who inject drugs (**a**, **c**) and HIV+ people who inject drugs (**b**, **d**) in Andalusia, Spain with various treatment scenarios and 95% Sustained Virologic Response

If treatment were scaled-up to both PLWH as well as the broader PWID population with HCV monoinfection, then greater impact could be achieved. For example, if all coinfected individuals were diagnosed and treated annually from 2020, and in addition 10% of HCV monoinfected PWID were treated annually, then this could lead to a reduction in the annual number of new diagnosed coinfections by 22% (95%I: 19–32%) (Fig. 2b, green line), an increase in the number of new HCV infections by a relative 31% (95%I: 26–38%) among HIV+ populations (Supplementary Figure 6), and a reduction in the number of new HCV infections overall by a relative 36% (95%I: 35–41%) 2015–2030 (Fig. 4b, green line).

### Sensitivity analyses

**Fig. 5:**
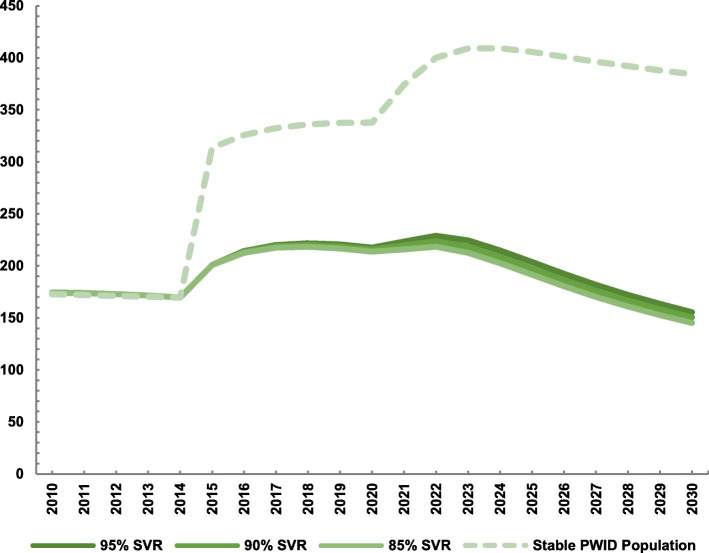
Mean model projections of the number of new HIV/HCV diagnosed individuals with all PLWH and 10% of HCV monoinfected PWID treated annually from 2020 among PWID and MSM between 2015 and 2030, comparing scenarios with varying sustained virologic response rates (solid lines) and a stable PWID population size (dashed line)

Sensitivity analyses on our scenario with all PLWH and 10% of HCV monoinfected PWID were treated annually from 2020 showed that the model projections were highly sensitive to assumptions regarding PWID population dynamics. If the PWID population remained constant and did not decline starting in 2010, it would result in a mean increase in the number newly diagnosed coinfections among PWID and MSM (from 313 in 2015 to 384 in 2030). This compares to a reduction in the number newly diagnosed coinfections among PWID and MSM at baseline (Fig. 5). Little differences were observed when reducing DAA SVR rates to 90% or 85%, which resulted in slightly fewer newly diagnosed coinfections due to less opportunity for reinfection (Fig. 5).

## Discussion

Our analysis indicates that recent scale-up of HCV treatment to HIV/HCV coinfected individuals in Spain since 2015 may have had an impact on reducing the number of existing and newly diagnosed HCV infections among PLWH, but will not achieve microelimination among HIV/HCV coinfected individuals, or elimination more broadly. Further scale-up to diagnose and treat all PLWH each year could halve HCV incidence among PWID by 2030, but will likely not achieve the WHO elimination goal of 80% reduction in HCV incidence by 2030. Elimination or microelimination efforts should focus on HCV screening, linkage to care, treatment, and harm reduction provision among both coinfected individuals and the broader population at continued risk of transmission, such as PWID.

### Strengths and weaknesses

To our knowledge, this is one of the first analyses to evaluate the impact of HCV treatment programs prioritizing PLWH on achieving the WHO elimination targets among PLWH and the broader population. One previous study conducted in France modeled the HCV epidemic among HIV-positive patients from all risk groups, finding that elimination was feasible among these groups [16]. However, their study did not incorporate dynamic HCV transmission from the monoinfected HCV population, assuming a low fixed incidence among HIV-positive PWID. Due to a combination of biological, behavioral, and historical factors [62–64], the majority of PWID acquire HCV within the first few years of injecting initiation and prior to their HIV infection [65]. Hence, the incidence of acute HCV among HIV+ PWID is low and may underestimate the true risk of reinfection in many settings like Andalusia. Indeed, we found that the risk of reinfection and continued incidence of new HIV/HCV coinfections among PWID important drivers of the epidemic. A strength of our model, thus, is the use of a dynamic HIV and HCV coinfection model incorporating injecting transmission of HCV from outside the HIV/HCV coinfected population. Our work supports previous modeling analyses that scale-up of HCV treatment to PWID could have a substantial prevention benefit and reduce incidence at a population level [5–9], but highlights the limitations of focusing elimination efforts only on subsets of risk groups (such as HIV+ PWID). Additionally, our modeling findings are similar to analyses focusing on HCV transmission among MSM [24, 25], which have found that substantial HCV transmission from HIV-uninfected or undiagnosed MSM may hamper HCV elimination efforts if treatment is focused only on HIV-diagnosed individuals.

As a modeling study, our analysis has a number of limitations. Firstly, we are limited by uncertainty in underlying epidemiological data. To account for this uncertainty, we sampled the prevalence from wide uncertainty bounds and ran numerous simulations which fit to different epidemic scenarios and propagated this uncertainty in future projections. Despite this uncertainty, all our projections supported our general finding that HCV elimination among PWID is unlikely to be achieved through current treatment rates, and that it requires targeting both HIV/HCV-coinfected and HCV-monoinfected individuals. We additionally lacked data on HCV retreatment rates among HIV-infected PWID who were successfully treated and then reinfected, and assumed that reinfection were eligible for retreatment. We believe this is a reasonable assumption, as firstly, guidelines do not mention retreatment restrictions, and secondly, accelerated disease progression among HIV/HCV coinfected individuals provides a strong indication for treatment/retreatment. However, if retreatment were not allowed, we would expect to achieve less impact.

Second, our analysis focuses on the impact of HCV treatment among HIV-infected individuals on burden among HIV+ MSM and PWID, but only includes treatment as prevention benefits among PWID. Most incident HCV infections in Spain are among PWID, and the majority of HIV/HCV coinfected individuals having a history of injecting drug use [17]. Within the HERACULES cohort in Andalusia, only 3% of HIV/HCV coinfected individuals were MSM [17]. Although traditionally MSM have been the focus of many studies of acute HCV among HIV-infected individuals, this is because PWID usually acquire HCV early on in their injecting careers and prior to their HIV infection [54]. As such, our study primarily focused on treatment as prevention benefits among PWID who contribute to the vast majority of overall HCV incidence. Additionally, we assume transmission epidemics between PWID and MSM populations are distinct and non-overlapping, based on phylogenetic analyses in Europe indicating that HCV transmission among HIV+ MSM occurs separately from PWID, [66, 67]. However, some overlap has been noted among the PWID and MSM populations in Australia [68]. Further, we used country-level (for Spain) HCV reinfection incidence data from HIV+ MSM and pooled HCV primary incidence data from HIV+ MSM because Andalusia-specific data were unavailable. Therefore, molecular and epidemiological data on HCV transmission specific to MSM in Andalusia would strengthen this analysis and allow for quantification of the additional benefit on MSM transmission, and potential transmission between populations.

Third, our study focuses only on MSM and PWID populations, as this comprised < 91% of reported risks in the Andalusia cohort [17]. Less than 1% of individuals reported risk due to being blood donors, and the remainder had none of the above reported risks. Due to the very small number of blood-donor derived infections, we neglected this group as the risk of ongoing incidence or reinfection due to this transmission mode is negligible. As the risk of HCV transmission through heterosexual transmission is low [69, 70], we believe it is possible that these individuals may not have reported PWID or MSM risk. The inclusion of these individuals in our model as PWID or MSM would not affect our results in terms of relative reductions in incidence (which is our primary outcome), although it would mean that the absolute numbers in each category were slightly higher than we report.

Fourth, we model a scenario of 100% screening and treatment of PWID from 2018 to highlight the potential maximum impact of a comprehensive HCV treatment strategy, but acknowledge that this type of scale-up would be ambitious. In reality, there are a number of barriers to the implementation of such a comprehensive diagnosis and treatment program. Enhanced diagnosis rates among PWID would likely require expanded provision of testing in both harm reduction services and outreach to identify harder to reach individuals, using multiple testing modalities such as point of care diagnosis and noninvasive testing. After diagnosis, strong systems ensuring effective linkage to care are required, particularly among PWID who may experience periods of homelessness and incarceration which may disrupt these linkages. Indeed, incarceration has been associated with increased HIV and HCV risk among PWID globally, and hence could pose a challenge to HCV prevention, yet at the same time could be an opportunity for HCV diagnosis and treatment in the DAA era. Finally, the cost of current DAA therapies is still high, which could act as a barrier to immediate universal scale-up of HCV treatment. As Spain has recently relaxed the HCV restrictions regarding therapy and allowing for universal access, it remains to be seen whether this translates into substantial uptake among the broader PWID population.

Fifth, we do not account for the impact of scaled-up harm reduction on the HCV and HIV epidemics among PWID. A recent Cochrane systematic review and meta-analysis found that opiate substitution therapy and high coverage needle and syringe programs are effective at preventing HCV, by roughly 50% (for OST), 76% (for high coverage NSP in Europe) and 74% in combination [71]. Modeling studies have shown the substantial impact that combination harm reduction can have on HCV epidemics among PWID [72], and future work should examine the potential impact of combination treatment and harm reduction among HIV+ populations.

Lastly, we note that our dynamic model predicts epidemic dynamics using epidemiological data from 2010 to 2017, which is the most recent data available for Andalusia. More recent data are needed, as they would allow for model validation and confirmation that treatment scale-up is leading to observed reductions in HCV incidence or chronic prevalence, and to confirm whether Andalusia is on track for HCV elimination.

## Conclusions

In conclusion, microelimination of HCV among HIV+ PWID in Spain requires a focus on elimination of HCV transmission among PWID, both moninfected and coinfected alike. Eliminating HCV from HIV-positive populations will require efforts to prevent HCV infection among PWID prior to their HIV infection, and therefore elimination efforts should focus on combination prevention among the broader PWID population.

## Supplementary information

**Additional file 1: Figure S1.** Mean model projections of the (A) number of HIV/HCV diagnosed active PWID (B) number of new HIV/HCV diagnosed active PWID, (C) number of chronic HCV infections among active PWID, and (D) number of new chronic HCV infections among active PWID with observed scaled-up DAA treatment rates from 2015 in Andalusia with various treatment scale-up scenarios. **Figure S2.** Mean model projections of the number of new. HIV/HCV diagnosed MSM with observed scaled-up DAA treatment rates. From 2015 in Andalusia with various treatment scale-up scenarios. **Figure S3.** Model projections (mean and 95% intervals) for HCV chronic prevalence and incidence among people who inject drugs (A,C) and HIV+ people who inject drugs (B,D) in Andalusia, Spain with observed scaled-up DAA treatment rates from 2015. Black line indicates mean projection, grey lines indicate 2.5–97.5% interval projections. **Figure S4.** Comparison of the model projected trends in HCV chronic prevalence among HIV+ individuals with a history of injecting drug use against the data. Model projections shown as solid line (black the mean projection, and gray lines the 2.5–97.5% interval projections). Calibration data sampling bounds (minimum and maximum) shown in red. **Figure S5.** Comparison of the model projected trends in HIV and HCV prevalence against the data. HIV prevalence (A,B) and HCV chronic prevalence (C,D) shown among people who inject drugs by injecting duration (> 10 years and < 10 years). Model projections shown as solid lines (black the mean projection, and gray lines the 2.5–97.5% interval projections). Calibration data sampling bounds (minimum and maximum) shown in red. **Figure S6.** Mean model projections of the number of new HCV infections among HIV+ MSM and PWID in Andalusia, Spain if all coinfected individuals were diagnosed and treated annually from 2020, and in addition 10% of HCV monoinfected PWID were treated annually.

## Data Availability

All of the data sources used for this study are publicly available in published literature and cited in Table 1, with the exception of the HERACLES cohort data (PI: Antonio Rivero-Juarez). The HERACLES was supported by the Ministerio de Sanidad (RD12/0017/0012) integrated in the Plan Nacional de I + D + I, and cofinanced by the ISCIII-Subdirección General de Evaluación and the Fondo Europeo de Desarrollo Regional (FEDER), the Fundación para la Investigación en Salud (FIS) del Instituto Carlos III (PI15/01017), and the Red de Investigación en SIDA de España ISCIII-RETIC (grant number: RD16/0025/0034).

## Abbriviations

HCV: Hepatitis C virus
PLWH: People living with HIV
PWID: People who inject drugs
MSM: Men who have sex with men
DAAs: Direct-acting antiviral therapies
SVR: Sustained viral response
OST: Opiate substitution therapy
