# Supplementary material for: What is needed to achieve HCV microelimination among HIV-infected populations in Andalusia, Spain: a modeling analysis
Source: BMC Infect Dis. 2020 Aug 8;20:588. doi: 10.1186/s12879-020-05285-z (PMC7414743; doi:10.1186/s12879-020-05285-z)
Supplement: Supplementary file 1 — Additional file 1: Figure S1. Mean model projections of the (A) number of HIV/HCV diagnosed active PWID (B) number of new HIV/HCV diagnosed active PWID, (C) number of chronic HCV infections among active PWID, and (D) number of new chronic HCV infections among active PWID with observed scaled-up DAA treatment rates from 2015 in Andalusia with various treatment scale-up scenarios. Figure S2. Mean model projections of the number of new. HIV/HCV diagnosed MSM with observed scaled-up DAA treatment rates. From 2015 in Andalusia with various treatment scale-up scenarios. Figure S3. Model projections (mean and 95% intervals) for HCV chronic prevalence and incidence among people who inject drugs (A,C) and HIV+ people who inject drugs (B,D) in Andalusia, Spain with observed scaled-up DAA treatment rates from 2015. Black line indicates mean projection, grey lines indicate 2.5–97.5% interval projections. Figure S4. Comparison of the model projected trends in HCV chronic prevalence among HIV+ individuals with a history of injecting drug use against the data. Model projections shown as solid line (black the mean projection, and gray lines the 2.5–97.5% interval projections). Calibration data sampling bounds (minimum and maximum) shown in red. Figure S5. Comparison of the model projected trends in HIV and HCV prevalence against the data. HIV prevalence (A,B) and HCV chronic prevalence (C,D) shown among people who inject drugs by injecting duration (> 10 years and < 10 years). Model projections shown as solid lines (black the mean projection, and gray lines the 2.5–97.5% interval projections). Calibration data sampling bounds (minimum and maximum) shown in red. Figure S6. Mean model projections of the number of new HCV infections among HIV+ MSM and PWID in Andalusia, Spain if all coinfected individuals were diagnosed and treated annually from 2020, and in addition 10% of HCV monoinfected PWID were treated annually. [file 12879_2020_5285_MOESM1_ESM.docx]

**SUPPLEMENTARY INFORMATION**

**Supplementary Figure 1: Mean model projections of the (A) number of HIV/HCV diagnosed active PWID (B) number of new HIV/HCV diagnosed active PWID, (C) number of chronic HCV infections among active PWID, and (D) number of new chronic HCV infections among active PWID with observed scaled-up DAA treatment rates from 2015 in Andalusia with various treatment scale-up scenarios.**

**
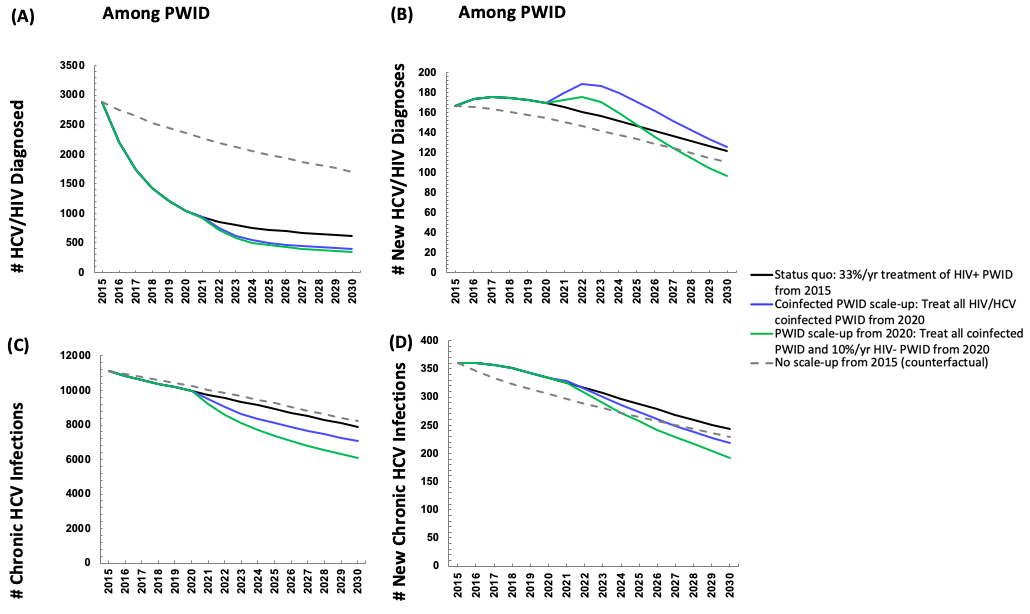
**

**Supplementary Figure 2: Mean model projections of the number of new**

**HIV/HCV diagnosed MSM with observed scaled-up DAA treatment rates**

**from 2015 in Andalusia with various treatment scale-up scenarios.**

**
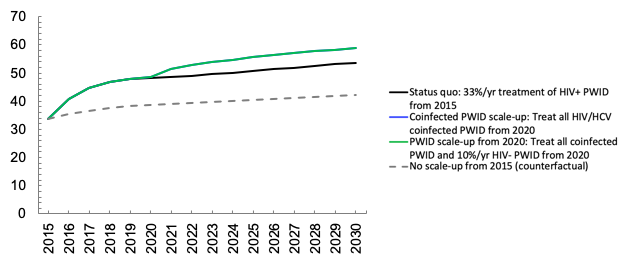
**

**Supplementary Figure 3: Model projections (mean and 95% intervals) for HCV chronic prevalence and incidence among people who inject drugs (A,C) and HIV+ people who inject drugs (B,D) in Andalusia, Spain with observed scaled-up DAA treatment rates from 2015.** Black line indicates mean projection, grey lines indicate 2.5-97.5% interval projections.

**
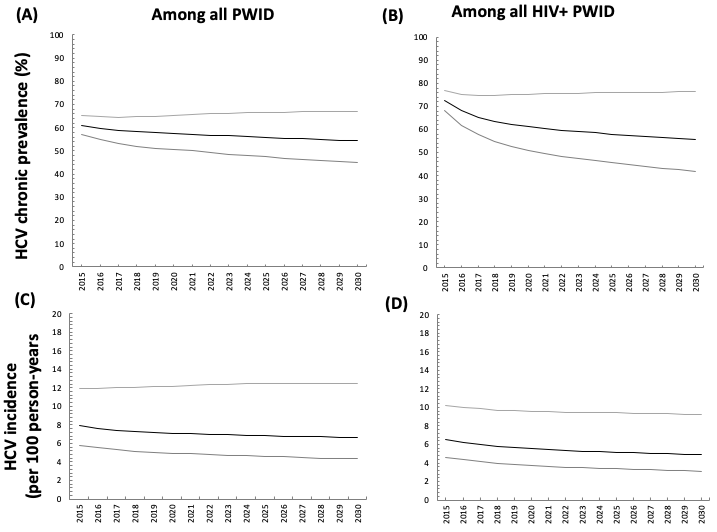
**

**Supplementary Figure 4: Comparison of the model projected trends in HCV chronic prevalence among HIV+ individuals with a history of injecting drug use against the data.** Model projections shown as solid line (black the mean projection, and gray lines the 2.5-97.5% interval projections). Calibration data sampling bounds (minimum and maximum) shown in red.

**Supplementary Figure 5: Comparison of the model projected trends in HIV and HCV prevalence against the data.** HIV prevalence (A,B) and HCV chronic prevalence (C,D) shown among people who inject drugs by injecting duration (>10 years and <10 years). Model projections shown as solid lines (black the mean projection, and gray lines the 2.5-97.5% interval projections). Calibration data sampling bounds (minimum and maximum) shown in red.


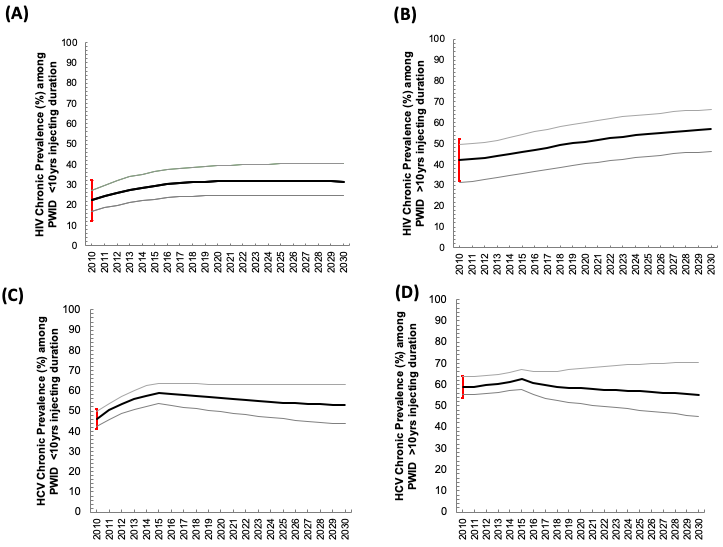


**Supplementary Figure 6: Mean model projections of the number of new HCV infections among HIV+ MSM and PWID in Andalusia, Spain if all coinfected individuals were diagnosed and treated annually from 2020, and in addition 10% of HCV monoinfected PWID were treated annually.**

**Supplementary methods**

**Data estimate for HCV primary incidence among HIV+ MSM.** There is one published study of HCV primary incidence among HIV+ MSM from Andalusia, from a prospective cohort of HIV infected individuals in 2006. In this study, primary incidence among HIV+ MSM was 0.19 per 100 person-years [95%CI 0.13-0.47](1). We elected not to use these data given the age of this data, because the estimate is far lower than observed in other European settings at that time and increases in HCV incidence were observed widely across Europe after 2006 (although we acknowledge increases were more pronounced in Western Europe compared to Southern Europe)(2), and because this primary incidence estimate was not compatible with current estimates of the number of HIV/HCV coinfected MSM in Andalusia unless incidence was very high prior to 2006 which was not felt to be more likely than a higher incidence in recent years. As such, for the model we use a pooled global incidence estimate among HIV+ MSM from a global systematic review and meta-analysis, of 1.09 (95% 0.73-1.61) from 2010, consistent with estimates from Southern Europe which, depending on the statistical methods used, estimate a mean incidence of 1-2/100 person-years in 2010(3). If the true incidence is lower than we assume, the impact of treatment on HCV prevalence among HIV+ MSM would be more than we estimate.

**References**

1. Palacios R, Mata R, Aguilar I, Muñoz L, Ríos MJ, Vergara S, et al. High seroprevalence but low incidence of HCV infection in a cohort of patients with sexually transmitted HIV in Andalusia, Spain. J Int Assoc Physicians AIDS Care (Chic). 2009;8(2):100-5.

2. van Santen DK, van der Helm JJ, Del Amo J, Meyer L, D'Arminio Monforte A, Price M, et al. Lack of decline in hepatitis C virus incidence among HIV-positive men who have sex with men during 1990-2014. J Hepatol. 2017;67(2):255-62.

3. Hagan H, Jordan AE, Neurer J, Cleland CM. Incidence of sexually transmitted hepatitis C virus infection in HIV-positive men who have sex with men. AIDS (London, England). 2015;29(17):2335-45.
